# A Systematic Review of Antibiotic Prescription for Acute Otitis Externa

**DOI:** 10.7759/cureus.14149

**Published:** 2021-03-27

**Authors:** Zahir Mughal, Ramanathan Swaminathan, Husham B Al-Deerawi, Scott Henney, Richard Bickerton

**Affiliations:** 1 Otolaryngology, Warwick Hospital, Warwick, GBR

**Keywords:** antibiotic prescription, otolaryngology, primary care, otology, acute otitis externa

## Abstract

Background: There is a perception among ear, nose, and throat (ENT) surgeons that oral antibiotics are over-prescribed for acute otitis externa (AOE), and the potential for topical therapy as first-line treatment is not fully realized in primary care. We evaluated the prescription rate of topical and oral antibiotics for AOE in primary care and in patients referred to the ENT emergency clinic.

Methods:* *A systematic review was performed by searching the MEDLINE, Embase, PubMed, and Google Scholar databases between January 1990 and October 2020. The quality of the evidence was assessed using the Grading of Recommendations, Assessment, Development, and Evaluations (GRADE) tool. The outcome measures were the rate of topical and oral antibiotic prescriptions for AOE by primary care practitioners and the rate of oral antibiotic prescriptions that were not indicated.

Results: Seven studies met the inclusion criteria. The overall quality of evidence was moderate. The rate of topical antibiotic prescriptions was between 77% and 95%, and oral antibiotics varied between 6% and 30% in patients that were managed only in primary care. Patients that were referred to an ENT emergency clinic had initially been treated by primary care practitioners with topical antibiotics in 14%-60% of cases and oral antibiotics in 16%-17%. The most commonly prescribed oral antibiotics were Amoxicillin and Co-amoxiclav (amoxicillin/clavulanic acid). No study had comprehensively reviewed the indication for oral antibiotics.

Conclusion: The rate of topical antibiotic prescriptions for AOE was high in primary care; however, the rate was frequently suboptimal in patients attending the ENT emergency clinic. Although the rate of oral antibiotic prescriptions was relatively low, the choice of antibiotic for empirical treatment frequently did not cover the typical bacteria in AOE. There is a need for improvement in primary care prescribing of topical therapy prior to referral to the ENT emergency clinic.

## Introduction and background

Acute otitis externa (AOE) is a common problem for general practitioners (GPs) [1] and is one of the top 10 reasons for antibiotic prescriptions in general practice [2]. It is also the condition most frequently referred to the ear, nose, and throat (ENT) emergency clinic [3]. The most effective treatment for uncomplicated AOE is topical antibiotics, achieving clinical cure rates of up to 80% within 10 days of therapy [4]. Two meta-analyses [4,5] have concluded that the choice of topical antimicrobial is not important as the impact of different preparations on the rates of clinical and bacteriologic cure is minimal. Oral antibiotics, however, are associated with disease persistence and recurrence in mild cases [1] and are therefore a poor choice for initial therapy. 

Inappropriate systemic antibiotic prescriptions can give rise to antibiotic resistance, which is associated with difficult-to-treat infections and carries an increased risk of mortality [6]. The United Kingdom (UK) government’s five-year national action plan from 2019 to 2024 [6] to control antimicrobial resistance emphasizes the role of guidelines and improves education in order to cut down on inappropriate antibiotic prescriptions. 

The UK National Institute for Health and Care Excellence (NICE) guidelines on antimicrobial prescribing [7], last updated in 2020, advocate treatment with a topical antibiotic with or without a steroid. NICE [7] also recommends that oral antibiotics, such as flucloxacillin, are indicated only in the presence of systemic signs of infection or cellulitis extending outside of the ear canal. The American Academy of Otolaryngology-Head and Neck Surgery Foundation (AAO-HNSF) published its updated guidelines in 2014 [8]. The primary goal of this guideline was to promote the appropriate use of antibiotics. They explicitly stated that systemic antibiotics should not be prescribed as initial therapy for uncomplicated AOE unless there is an extension beyond the ear canal or in the presence of specific host factors (diabetes, immunocompromised state, and previous radiotherapy) [8].

Treatment failure due to unnecessary use of oral antibiotics and a lack of topical therapy is common in our experience of reviewing patients with uncomplicated AOE that have been referred by their GPs to the ENT emergency clinic. This has several implications including undue pressure on the secondary care service, prolonged patient suffering, cost, potential adverse drug effects, and risk of antimicrobial resistance. We therefore evaluated the rate of oral and topical antibiotic prescriptions for uncomplicated AOE in primary care by performing a systematic review of the published literature.

## Review

Methods

This systematic review was performed according to the Preferred Reporting Items for Systematic Reviews and Meta-Analyses (PRISMA) statement standards [9]. The review protocol was not registered with the International Prospective Register of Systematic Reviews (PROSPERO) database.

Outcome Measures

The primary outcome measures were the rates of topical, oral, or a combination of antibiotic prescriptions for uncomplicated acute otitis externa (AOE) in primary care. The secondary outcome measures included the choice of prescribed antibiotics and the rate of oral antibiotic prescriptions without a valid indication. 

Literature Search Strategy

The electronic databases of MEDLINE, Embase, PubMed, and Google Scholar were searched. The last search was run on October 25, 2020. The MEDLINE and Embase search terms were ‘(antibiotics OR antibacterial agents OR topical OR treatment OR therapeutics) AND (primary care OR primary healthcare OR general practice OR general practitioner OR family practice) AND (otitis externa).’ The Google Scholar search was ‘"otitis externa" + antibiotics|treatment|therapeutics|topical + "general practice"|"primary care"|"general practitioner"|"family practice" - media - chronic - fungal - viral - dermatitis - allergic - animal.’ The PubMed search was ‘((therapeutics) OR (treatment) OR (antibiotics) OR (topical)) AND (otitis externa) AND ((general practice) OR (general practitioner) OR (primary care) OR (family practice)).’ Related articles on PubMed, ‘cited by’ feature on Google Scholar, and article bibliographies were also used to identify articles. Pre-prints prior to peer review were also included via Authorea.

Eligibility Criteria

Inclusion criteria were publications between January 1990 and October 2020, English language, humans, all age groups, and patients with AOE. Exclusion criteria included hospital prescriptions as we were interested in only general practice prescribing patterns. Patient with atypical infections such as viral or fungal or underlying dermatoses or chronic conditions were excluded to avoid skewing the sample to difficult-to-treat cases.

Study Selection

Articles were screened by titles and abstracts. Full texts of relevant articles were retrieved and independently assessed by two authors (ZM and RS). Quality assessment of studies was performed independently by both authors. Consultation was obtained from an independent third author (HB) for any discrepancies. 

Assessment of the Quality of Evidence

The GRADE (Grading of Recommendations, Assessment, Development, and Evaluations) framework [10] was used to assess the quality of the body of evidence. It is based on five categories of imprecision, indirectness, risk of bias, inconsistency, and publication bias [10]. Publication bias, however, tends not to be a problem in studies on prescription rates as these are non-comparative and do not report P values. GRADE has four levels of quality of evidence: very low, low, moderate, or high [10]. It is a widely adopted tool for rating the quality of evidence in systematic reviews and is designed to examine management strategies in healthcare [10].

Results

**Figure 1:**
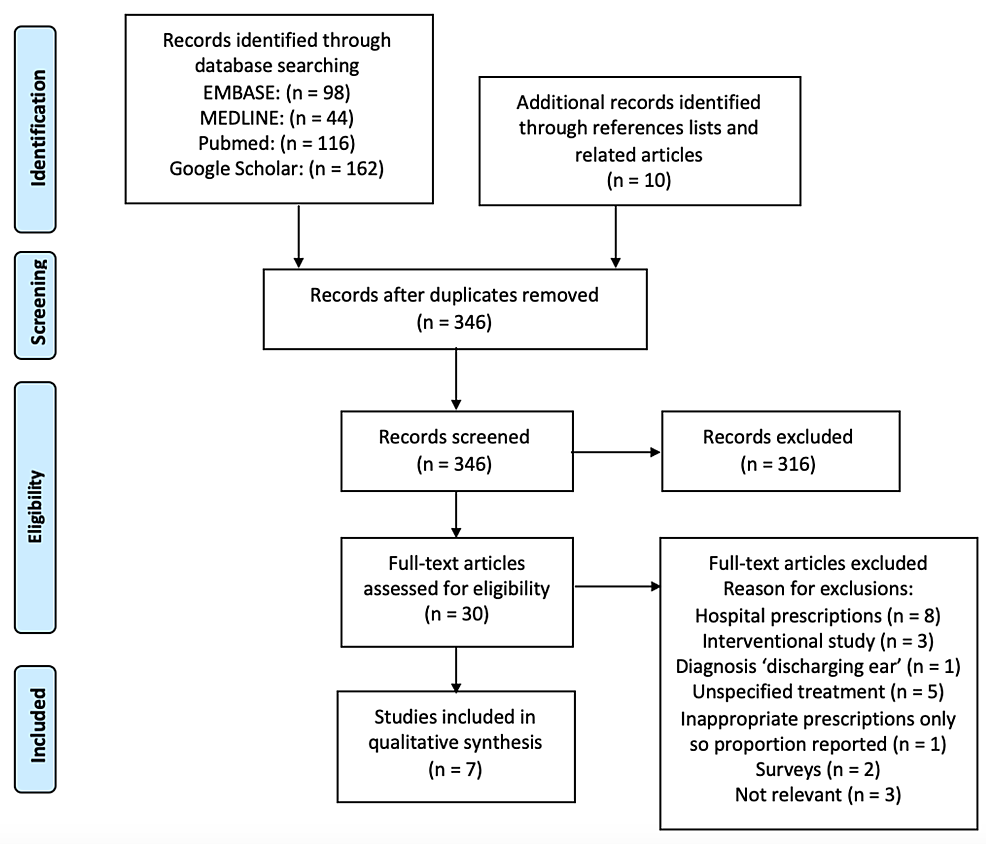
PRISMA flow diagram PRISMA, Preferred Reporting Items for Systematic Reviews and Meta-Analyses.

Our search strategy identified a total of 430 articles. This included results from Embase (n = 98), MEDLINE (n = 44), PubMed (n = 116), Google Scholar (n = 162), and other sources (n = 10), as shown in the PRISMA flow chart in Figure 1. After deduplication, 346 records were screened by title and abstract, of which 316 articles were found not to be relevant to our study question. Of the remaining 30 articles, review of full texts identified seven studies that were eligible for final analysis, in which 31,650 patients with otitis externa (OE) were assessed. A summary of our systematic review findings is presented in Table 1.

**Table 1:** Summary of included studies including rate of topical and oral antibiotic prescriptions for otitis externa in primary care (1990-2020) * These are the exact figures reported in the article by Rowlands et al. [1]. The percentages are based on the number of prescriptions and therefore correspond approximately but not exactly to the total number of OE patients. The percentages do not total 100%. Although the reason for this was not stated in their article, it may be due to repeated prescriptions used for the same patients as 6,515 patients had consulted more than once.

Study		Study design	Study setting	Number of OE patients	Findings n (%)		
					Topical antibiotics	Oral antibiotics	Combination of topical and oral
Rowlands et al., 2001 [1], UK		Retrospective observational	UK General Practice Research Database	30,412*	25,933 (85%)*	6,363 (21%)*	4,646 (15%)*
Selwyn and Lau, 2009 [11], UK		Retrospective observational	General practices (n = 2)	287	221 (77%)	17 (6%)	46 (16%)
Cheffins et al., 2009 [12], Australia		Retrospective audit	General practices (n = 8)	201	191 (95%)	61 (30%)	Not reported
Pabla et al., 2011 [13], UK		Prospective audit	ENT emergency clinic (n = 1)	106	15 (14%)	17 (16%)	30 (28%)
Trinidade et al., 2011 [14], UK		Prospective audit	ENT emergency clinic (n = 1)	100	26 (26%)	17 (17%)	33 (33%)
Tierney et al., 2001 [15], UK	Cycle 1	Prospective audit	ENT emergency clinic (n = 1)	38	19 (50%)	Not reported	Not reported
	Cycle 2			42	25 (60%)	Not reported	Not reported
Greer et al., 2018 [16], Thailand		Retrospective observational	Primary care units (n = 32)	464	Not reported	369 (80%)	Not reported

Topical Antibiotics

Rowlands et al. [1] analyzed a large UK general practice database and found that the prescription rate for topical therapy was 85%. The most commonly prescribed ear drops contained a combination of a steroid and an antibiotic (64%), followed by steroid alone (35%) and antimicrobials alone (2%). Gentisone HC (gentamicin 0.3%/hydrocortisone acetate 1%) drops (26%) were the most common steroid/antibiotic prescription, and dexamethasone was the most common steroid drop prescription (30%) [1]. Selwyn and Lau [11] examined two GP surgery records and identified a topical prescription rate of 77%. The most common topical prescription was Otomize (neomycin/dexamethasone/acetic acid) spray (58%), followed by Locorten-Vioform (flumetasone 0.02%/clioquinol 1%) spray (13%) [11]. Cheffins et al. [12] audited eight general practices from three cities in North Queensland, Australia. They found that 95% of patients were treated with ear drops, most commonly a steroid/framycetin preparation (48%), and Ciprofloxacin drops were used as second line in 31% of cases [12]. 

Three studies reviewed GP referrals to the ENT emergency clinic in three UK hospitals and examined primary care treatment of AOE. The topical antibiotic prescription rates were 14% [13], 26% [14], and 50% [15], respectively. The latter group, Tierney et al. [15], sent out letters to their local GPs encouraging them to use topical medication for AOE, and a re-audit found that the topical prescription rate had increased to 60% [15]. The most common topical agent was reported to be Locorten-Vioform (flumetasone 0.02%/clioquinol 1%) [14].

Oral Antibiotics

Rowlands et al. [1] found that the rate of monotherapy with oral antibiotics was 21%. The most frequent prescriptions were Amoxicillin or Ampicillin (34%), followed by Co-amoxiclav (amoxicillin/clavulanic acid) (19%), Flucloxacillin (15%), and macrolides (14%) [1]. Selwyn and Lau [11] found that 6% of patients were given only an oral antibiotic, usually Amoxicillin (11%). Cheffins et al. [12] found an oral antibiotic rate of 30%, but regional variation between three cities in North Queensland, Australia, was noted (16%-37%). A northern Thailand study [16] of oral antibiotic prescriptions in 32 primary care units identified that 80% were treated with oral antibiotics. 

Of the hospital-based studies, Pabla et al. [13] found that GPs prescribed oral antibiotics in 16% of patients that had attended their ENT emergency clinic. Trinidade et al. [14] showed a similar rate of 17%, often with Co-amoxiclav (amoxicillin/clavulanic acid), followed by Amoxicillin and Erythromycin. 

Combination of Topical and Oral Antibiotics

Rowlands et al. [1] found that the rate of combination therapy with topical and oral antibiotics was 15%. Selwyn and Lau [11] identified a similar rate of 16%. Pabla et al. [13] and Trinidade et al. [14] found that the rate was higher at 28% and 33%, respectively. 

Unnecessary Oral Antibiotic Prescriptions

Only one study by Trinidade et al. [14] had reviewed the indication for oral antibiotics prescribed for AOE in patients attending the ENT emergency clinic. They identified that oral antibiotics, either given in combination or alone, were started by GPs in 50% of patients [14]. They deemed oral antibiotics were only indicated in 10 patients to treat perichondritis [14]. Three of these patients were not even prescribed oral antibiotics [14]. This infers an unnecessary oral antibiotic prescription rate of 86%.

Discussion

Summary of Main Findings

Topical antibiotics: The rate of topical antibiotic prescriptions in primary care was 77%-95%. The rate of topical antibiotics prescribed by GPs for patients that were referred to the ENT emergency clinics varied widely from 14% to 50% and up to 60% after an educational intervention for GPs. The wide variation observed in patients attending the emergency clinics may be related to the local referral criteria for acceptance into the clinic, which often vary between ENT departments.

Oral antibiotics: The prevalence of prescriptions for oral antibiotics as monotherapy was 6%-30% in patients treated in primary care. A narrower primary care prescription rate of 16%-17% was observed in patients attending the ENT emergency clinic. When oral antibiotics were prescribed, Amoxicillin and Co-amoxiclav (amoxicillin/clavulanic acid) were consistently chosen by GPs to treat otitis externa. The use of oral antibiotics as monotherapy may be an estimate of the rate of cases of otitis externa that were suboptimally managed as the inferred omission of topical antibiotics highlights a potential unawareness among prescribers that topical therapy is the mainstay of treatment. The Thailand study by Greer et al. [16] found an oral antibiotic prescription rate of 80%; however, this was considered an outlier as described below. Trinidade et al. [14] suggested that the rate of unnecessary oral antibiotics, which were defined as lacking a valid indication, may be as high as 86% according to their dataset. However, this finding should be treated with caution, also discussed below.

Comparison With Existing Literature

This is a rare review to examine the prescribing pattern of primary care practitioners for AOE. We identified a number of survey studies of GPs’ self-perceived prescribing preference, which generally over-stated the use of topical prescription compared to our findings. Robertson and Bennett [17] conducted a survey of 51 military GPs in the catchment area of the British Military Hospitals at Rinteln and Hannover, Germany. They found a high preference for topical antibiotics and steroid drops (89%) [17]. Tierney et al. [15] conducted a survey of 173 GPs in London, UK, and found that a high proportion (84%) stated that they would use topical medication as first-line treatment. Mildenhall et al. [18] conducted a United States of America (US) regional survey of 117 primary care clinicians to assess adherence to clinical practice guidelines. Treatment with topical therapy was reported by 94% of clinicians, and avoidance of systemic antibiotics was reported by 84% [18]. Cheffins et al. [12] conducted a survey of 39 GPs from three cities in North Queensland, Australia, which found oral antibiotics were prescribed by 23%. The respondents stated that they preferred topical treatment, with 50% stating they used Framycetin/steroid and 26% stating Ciprofloxacin [12].

The frequent use of Amoxicillin and Co-amoxiclav (amoxicillin/clavulanic acid) identified in our review was concerning as the causative bacteria in AOE are predominantly Pseudomonas aeruginosa and Staphylococcus aureus [8], and these antibiotics are inactive against these pathogens. Ciprofloxacin and Flucloxacillin are better choices when indicated. A UK study of general practice electronic healthcare records of 72,278 patients with a diagnosis of otitis externa between 2010 and 2015 found that the rate of inappropriate choice of oral antibiotics was high at 67% [19]. They also found that the most commonly prescribed inappropriate antibiotic was Amoxicillin [19]. The findings of this study and our results indicate that the inappropriate selection of oral antibiotics in AOE appears to be a highly prevalent problem in primary care in the United Kingdom. Not only ineffective, this practice may also lead to avoidable adverse drug events, non-compliance, antibiotic resistance, and the associated healthcare costs [5,8]. Topical antibiotics do not typically suffer with these problems [8]. The high local concentration of topical antibiotics far exceeds the minimum inhibitory concentrations needed to eradicate even resistant organisms [8]. This may explain the equivalent efficacy of various topical preparations demonstrated in meta-analyses [4,5].

Quality of the Evidence

Indirectness: The study by Rowlands et al. [1] had a large sample size of the UK primary care population with otitis externa. Their data was obtained from a primary care database, which is considered broadly representative of the UK population in terms of sex, age, and ethnicity [19]. This facilitates generalizability and applicability of their findings. The other UK-based general practice study by Selwyn and Lau [11] stated that they included all patients including those with immunocompromising conditions. Both of these studies analyzed an unfiltered sample of patients with otitis externa in primary care and therefore provided a representative depiction of real-life prescribing patterns for varying degrees of severity of otitis externa and host factors. However, the lack of stratification of their cases into uncomplicated and complicated cases impeded the evaluation of whether the oral antibiotic prescriptions were indicated, which was one of our study objectives. Unfortunately, all the studies shared this limitation.

Risk of bias: The Thailand study [16] of primary care units was at high risk of bias in selecting severe disease as patients were identified by the presence of pyrexia. This may explain the high rate (80%) of oral antibiotics in this study [16]. These prescriptions were indicated and therefore deemed appropriate. The three hospital audit studies [13-15] were also a highly selected sample of patients that were referred to the ENT emergency clinic. These patients may have represented severe disease that had failed treatment in the community or were poorly managed cases in primary care. There is some evidence to suggest the latter as 24% [14] to 42% [13] of patients were noted to have had no treatment prior to attendance to the ENT emergency clinic. Although the findings from these three hospital audits were not representative of the general GP population, they did reflect the cases that tend to attend ENT clinics.

Imprecision: All the studies except the study by Rowlands et al. [1] were limited by small sample sizes. Trinidade et al. [14] had provided some data to indicate that the rate of unnecessary oral antibiotic prescriptions was as high as 86%. However, the group had only used the diagnosis of perichondritis as an indication for oral antibiotics. The number of patients with an immunocompromised status or systemic upset were not accounted for, and therefore we cannot reliably deduce the rate of unnecessary oral antibiotic prescriptions. No other study had evaluated the validity of the oral antibiotic prescriptions. 

Inconsistency: A high degree of variation in the rates of topical and oral antibiotic prescriptions was found in the five UK-based studies [1,11,13-15]. This suggests that there may be regional variation in prescribing practice. The Australian study [12] also identified regional variation of oral antibiotic prescriptions between three cities. The Thailand study [16] was deemed an outlier due to significant heterogeneity in patient population and healthcare resources, compared to the other studies included in our review, and therefore excluded from our summary of findings.

Overall GRADE assessment: Our findings on rate of oral and topical antibiotic prescriptions as well as choice of antibiotics were generally applicable to GPs in the United Kingdom and probably to other similarly developed countries, but several limitations impaired the overall quality of evidence resulting in a moderate rating. The quality of evidence in relation to the rate of unnecessary oral antibiotic prescriptions, which lacked a valid indication, was low and could not be reliably evaluated.

Implications for Practice

The ENT emergency clinic is an established component of most ENT units in the UK [20,21]. Referrals from primary care are often accepted by junior doctors, and it is important to ensure junior doctors are trained and guided on how to triage referrals to avoid overburdening the clinic [20,21]. Our review has highlighted that a significant proportion of patients attending the ENT emergency clinic were not commenced on topical therapy prior to secondary care review. Our review has therefore identified a need for greater primary care adherence to both the NICE and AAO-HNSF guidelines, which recommend topical therapy as first-line treatment for AOE [7,8]. Failure to respond to treatment is an indication for referral to the ENT emergency clinic. Commencement of topical therapy in primary care should therefore be a pre-requisite to review in the ENT emergency clinic. There are exceptions, such as problems related to the delivery of drugs, e.g., debris or canal edema, which may need to be addressed earlier in secondary care with microsuction or wick insertion [8]. 

Tierney et al. [15] conducted a survey of 173 GPs in London, UK, and found that 84% stated that they would use topical medication, but if the tympanic membrane could not be visualized, this figure dropped to 67%. In the presence of a perforation, only 45% of GPs would be happy to prescribe topical therapy [15]. This may be due to concerns related to ototoxicity and may explain the reluctance of some GPs to prescribe topical therapy in our review findings, particularly for patients that they were referring to secondary care. When taking referrals for otitis externa from GPs, ENT doctors can provide reassurance by citing the consensus statement published by ENT-UK stating that topical aminoglycosides can be used in the presence of a perforation or patent grommet for up to two weeks [22]. Alternatively, topical quinolone preparations, such as Ciprofloxacin, are non-ototoxic [23] and may further ease the concerns of primary care practitioners. Such discussions at the point of the primary and secondary care interfaces may serve as a valuable exchange between GPs and ENT doctors so that both can better understand expectations of each other.

Further Research

The current literature lacks studies that stratify AOE into complicated and uncomplicated cases, and therefore we were unable to determine the proportion of oral antibiotic prescriptions that lacked a valid indication. Hence, the current body of evidence is insufficient to estimate the rate of unnecessary oral antibiotic prescriptions in AOE. Due to the retrospective nature of the studies, we cannot be certain whether the low rate of primary care prescriptions for topical therapy observed in patients that were referred to the ENT emergency clinic was due to undocumented patient factors, such as occlusion of the canal with debris, or simply suboptimal primary care treatment. There is a need for well-designed prospective studies to address these problems. One study [15] demonstrated that simply sending out a letter to local GPs increased the use of topical therapy in primary care. Further research is also required in evaluating educational interventions to improve primary care prescribing for uncomplicated AOE.

## Conclusions

Topical antibiotics were predominantly prescribed for AOE in primary care considerably more often than oral antibiotics. However only a relatively small proportion of patients had received topical therapy in primary care prior to referral to the ENT emergency clinic. Insufficient evidence was available to determine the proportion of oral antibiotics prescribed by GPs that lacked a clinical indication. There was good evidence to indicate that GPs frequently prescribed an inappropriate oral antibiotic choice, such as Amoxicillin or Co-amoxiclav (amoxicillin/clavulanic acid), despite inefficacy of these antibiotics to the common bacteria in AOE.

This study draws attention to deficiencies in primary care initial management of AOE and the need for ENT doctors to reserve ENT emergency clinic hospital appointments for those patients that have been initiated on appropriate treatment in primary care. Educational initiatives should be directed to address these deficiencies.
